# A narrative review of anxiety regulation in PhD students based on Green model

**DOI:** 10.3389/fpsyg.2024.1351386

**Published:** 2024-07-09

**Authors:** Yue Ma, Ahui Yu, Haiqi Ma, Yang Zhao, Xiaojun Liu, Huimin Zhai, Yulin Gao

**Affiliations:** ^1^Macau University of Science and Technology, Taipa, Macao SAR, China; ^2^Southern Medical University, Guangzhou, Guangdong, China; ^3^Evidence Based Nursing and Midwifery Practice PR China: A JBI Centre of Excellence, School of Nursing, Southern Medical University, Guangzhou, China; ^4^The Eighth Affiliated Hospital, Sun Yat-sen University, Shenzhen, China

**Keywords:** PhD, anxiety, green model, psychological status, regulation

## Abstract

To gain a better understanding of the factors that contribute to anxiety among PhD students and the reasons for poor regulation in the current situation, this paper analyses the existing literature on anxiety among PhD students using Green's model. It also compares and evaluates various methods of regulating anxiety. The literature review to extract information on the causes and levels of anxiety, methods and outcomes of anxiety intervention and regulation, and to make comparisons. The study reveals that the phenomenon of PhD students' anxiety has intensified globally in recent years, especially after the end of the epidemic. PhD students experience anxiety due to research pressure, economic pressure, future development, and interpersonal pressure. The main influencing factors are currently the relationship with the supervisor, development prospects, social support, and peer comparison. Among the stress relief methods, the regulation of self-relaxation was found to have better effects on mild anxiety, such as positive thinking, meditation, yoga and physical exercise can be helpful for emotion release then help focus on problem solved. Whereas severe anxiety may require institutional and pharmacological support, also including using psychological therapy such as behavioral cognitive therapy and systematic desensitization methods. For university, competence to provide course-assisted guidance, such as writing groups, peer support, and time management, is also important. Academic communities should pay attention to the guidance on academic fairness. However, PhD students are often unaware of the resources available to them for coping with stress and may not take the initiative to seek psychological counseling or institutional assistance. Therefore, PhD students should receive support from various sources, be guided to express their thoughts, and receive additional education and academic assistance to manage stress. This will enhance their confidence and aid in improving their scientific research.

## 1 Introduction

In recent years, the number of PhD (Doctor of Philosophy) has increased rapidly. In 2018, China alone had a total of 389,518 PhD students (China National Bureau of Statistics, 2018; National Bureau of Statistics Database of China, 2018). There are plans to further expand the scale of doctoral enrollment in the future (Ministry of Education of the People's Republic of China., 2023). The countries with the highest number of PhD students are the U.S., Germany, and the U.K. Until 2020, there were more than 281,360 DRs enrolled in these three countries (Hazell et al., 2020). There has been a rising prevalence of anxiety and depression among PhD the whole world. The PhD community plays a crucial role in higher education and its outcomes (Schmidt and Hansson, 2018), with PhD programs closely linked to scientific research output (Wei, 2015). Compared to master's degree students, PhD students experience more severe anxiety (Divaris et al., 2012). Studies have indicated PhD have high levels of anxiety the whole world, especially among medical PhD students, particularly in clinical fields. Pressures stem from academic tasks, future career prospects, and publication demands (Mao, 2014).

Although the academic settings and society have adopted many ways to alleviate the pressure of doctoral students, such as the introduction of psychological adjustment public welfare courses, psychological counseling and psychological crisis practice defense system, corresponding scientific research writing courses, social subsidies for doctoral students and employment policies for talents. But at present, the anxiety of PhD students has not been alleviated, and even the prevalence of anxiety is more serious. Therefore, this paper uses Green's model for analysis. The Green model, specifically the PRECEDE-PROCEED model, has been effectively used in the field of health promotion evaluation (Didehvar et al., 2016; Payne et al., 2016). PRECEDE integrates predisposing, enabling, and reinforcing factors affecting target behaviors, while PROCEED supports policies, regulations, and environmental factors during educational interventions (Wang et al., 2018). Based on the Green's model, this paper analyzes influencing factors from PRECEDE and evaluates existing intervention programs from PROCEED to offer guidance for universities and individuals in addressing PhD anxiety. It makes the analysis of influencing factors and intervention effects include multiple levels, so as to have a more comprehensive understanding of doctoral anxiety.

The literature review involved searching for studies related to anxiety among PhD students. This was conducted across several databases, including PubMed, Web of Science, and ProQuest for English-language studies, and Wanfang, VIP, and CNKI for Chinese-language studies. We searched the literature using the following keywords: (‘anxiety' AND ‘PhD’ AND ‘regulation’) from inception to March 2024. Based on PRISMA 2020 Identification of studies via databases, finally we included 48 (Supplementary Figure 1). Among the included literatures, there were cross-sectional studies, intervention research and qualitative interviews (Supplementary Table 1). Covering specific cases and empirical evidence can strengthen the discussion and provide real-life examples to illustrate the breadth and comprehensiveness of Green's model in PhD students' anxiety.

## 2 PRECEDE stage: assessment of PhD students' anxiety

### 2.1. Social and environmental assessment

Social and environmental factors contributing to the anxiety experienced by PhD students encompass various dimensions. ① The surge in postgraduate enrollments and the pursuit of professional advancement have intensified the pressure to publish in high-quality journals (Liu et al., 2019). The escalating demand has strained the availability of core journals, rendering the publication process more challenging. In the context of China, the decade from 2009 to 2018 witnessed a 48% increase in national PhD enrollment, while the publication volume in Chinese Social Sciences Citation Index (CSSCI) journals declined by 22.65%. Approximately 80% of the 567 journals examined showed a downward trend in publication volume, with 48% experiencing a decrease of over 20% (Wang, 2022). ② The nature of PhD work compounds anxiety due to the absence of timely positive feedback, the monotonous repetition of tasks, and prolonged periods of experimentation without favorable results. Limited vacation time, coupled with an intense desire for success, leads some PhD students to forego holidays, engaging in overtime work. For instance, more than a third (35%) of respondents said their projects did not meet their initial expectations; 70% said they spent more than 40 h a week on their projects (Woolston, 2022). ③ A lack of comfort, minimal leisure, and limited engagement in social activities contribute to the PhD students' anxiety (Hazell et al., 2020). The absence of opportunities to share their concerns with others exacerbates negative emotions (Guo, 2019). ④ Interpersonal relationships and social isolation play a significant role in inducing academic burnout among PhD students. Strained relationships with teachers and peers, coupled with a lack of emotional support, heighten the risk of burnout (He et al., 2020). ⑤ Economic pressures arise from the absence of working income for full-time PhD candidates, reduced income for those pursuing on-the-job PhD's and inadequate rewards for scientific research. The economic disparity compared to peers who have entered the workforce contributes to a pervasive “sense of failure.” (Bazrafkan et al., 2016; Charles et al., 2022) ⑥ Peer pressure, conflicts, and negative feedback from colleagues can lead to distraction from scientific research. Unconscious comparisons and consolation may exacerbate feelings of incompetence and anxiety (Zhong et al., 2011; Hazell et al., 2020). ⑦ Cultural factors contribute to marriage anxiety among single female PhD students. Limited psychological support institutions, coupled with societal reluctance to seek help, exacerbate the challenges faced by these students (Guo, 2019). ⑧ Employment factors, including a pervasive “whether high or low is not enough” mentality, and uncertainty about job opportunities in the aftermath of the epidemic, contribute to the involution of PhD students' perceptions of their career prospects (Wang, 2016; Woolston, 2020a,b, 2022). ⑨ Family factors, encompassing marital considerations and traditional family cognitive factors, influence emotional regulation. The educational background and occupation of the mother, along with family and social status, impact PhD students' emotional well-being (Feng and Zhang, 2017). ⑩ Poor time management, exacerbated by additional tasks beyond scientific research, further compounds anxiety among PhD students. The inability to meet set tasks intensifies the already substantial pressure associated with scientific research (Wang, 2016; Wang et al., 2019).

### 2.2 Epidemiology and behavioral diagnosis

#### 2.2.1 Epidemiological diagnosis and characteristics: for number of anxiety PhD

A global consensus acknowledges an increasing prevalence of anxiety among doctors worldwide. A comprehensive survey conducted by Nature in 2019 encompassed 6,300 PhD students from various corners of the world. The findings revealed a 72% satisfaction rate among international PhD students, with 36% seeking assistance for anxiety or depression linked to their PhD pursuits (Woolston, 2019). In contrast, only 55% of PhD students in China expressed satisfaction with their PhD careers, with 40% seeking help for anxiety or depression stemming from academic challenges. Notably, studies in China underscore a high prevalence of anxiety among medical doctors. For instance, an anxiety detection rate of 24.76% was observed among 315 medical doctors (Tan and Chen, 2018). However, this detection rate appears to underestimate the actual prevalence. Epidemiological surveys further illuminate variations in anxiety levels among PhD students based on factors such as age, undergraduate specialization, gender, educational background, urban-rural origin, academic discipline (social science vs. natural science), and the classification of universities (e.g., key universities under the “985 Project,” “211 Project,” and “Double First-Class” initiative) from which PhD students graduate (Wu, 2019).

#### 2.2.2 Behavioral diagnosis: for stressors and coping skills

A pivotal aspect of behavioral diagnosis involves understanding the stress and coping processes that individuals undergo when confronted with challenges or failures. When faced with stressors, individuals typically employ three primary coping strategies—problem solving, emotional management, and avoidance. Problem-solving entails directly addressing and resolving issues, such as writing papers or completing tasks. In instances where direct resolution proves elusive, individuals' resort to emotional management, employing psychological defense mechanisms and cognitive adjustments. Seeking advice and external assistance may be part of this strategy. When emotional management fails, some individuals may resort to avoidance, as evidenced by a study where 37% of students in a vocational DPH project contemplated giving up (Hlabse et al., 2016). In severe cases, individuals may experience a sense of entrapment and exhibit suicidal tendencies. Physiologically, adverse responses trigger a triple reaction involving endocrine changes (e.g., adrenal gland enlargement), immune system alterations (e.g., thymic gland degeneration), and digestive system impacts (e.g., gastric erosion formation) (Tachè, 2014). The psychological manifestations include anxiety and withdrawal, hindering effective problem-solving and exacerbating the source of pressure, leading to further damage. Consequently, poor coping mechanisms can result in a state of physical and mental collapse, often accompanied by anxiety that may evolve into depression. Behind anxiety often lies the potential for depression, which may manifest in atypical forms such as “hidden depression.” Individuals experiencing “high-functioning depression” or “smiling depression” may outwardly function well socially while harboring internal struggles. This form of depression is challenging to detect due to its seemingly normal social functioning.

In the cognitive and response processes, Hans Selye's stress and adaptation theory delineates stress feelings into alert, resistance, and exhaustion periods. Failure to manage stress during the resistance period in the PhD stage may lead to negative responses during the exhaustion period, potentially resulting in severe consequences. Richard S. Lazarus's three-stage cognitive evaluation further emphasizes the importance of cognitive assessments at different stages, including primary evaluation, secondary evaluation, and re-evaluation. Given the advanced cognitive capabilities of PhD students, their anxiety often roots from continuous negative feedback in the cognitive evaluation process. For instance, the high-level task of publishing an article with its lengthy cycle can be emotionally taxing, particularly when manuscripts face multiple rejections. In the face of repeated setbacks, PhD students may develop learned helplessness and anticipatory grief, diminishing motivation for persistence and efforts. This cascade of challenges may lead to escalating anxiety, pushing individuals into a stage of failure accompanied by severe physical and mental reactions, and potentially contributing to the “pretender syndrome” involving self-denial (Inouye, 2021).

#### 2.2.3. Important behaviors and relatively unimportant behaviors

When confronting stressors that cannot be eliminated, the focus shifts to changing cognition and actions in the coping process. For significant behaviors, the emphasis lies in attitudinal shifts, such as redefining professional identity, reassessing the significance of reading, and confronting challenges for a deeper understanding. Simultaneously, breaking cognitive limitations and building self-confidence, enhancing metacognitive strategies becomes crucial. Metacognitive strategies mitigate learning disruptions arising from reluctance to draft or modify writing, fostering smoother academic progress and minimizing self-doubt. In contrast, for relatively unimportant behaviors, once self-confidence is established and a positive psychological learning will is generated, actions may include enhancing learning and research skills, adopting effective coping strategies, engaging in physical exercise, and managing interpersonal relationships, particularly within the academic “mentor, team, and laboratory” framework (Tan and Chen, 2018).

#### 2.2.4. High variability behaviors and low variability behaviors

Addressing sources of pressure with high variability or low variability poses challenges for immediate change or removal. Consequently, emphasis is placed on cognitive evaluation and coping strategies. High-variability approaches involve active participation in activities, seeking external assistance, and altering lifestyle and thought patterns, encompassing aspects like time management, cognitive enhancement, and improvement of research abilities (Meehan et al., 2023). Low-variability behavior is characterized by an internal aversion to unfamiliar circumstances, resistance to changing original ideas, and avoidance of tackling challenging problems through thoughtful consideration (Murguía Burton and Cao, 2022).

#### 2.2.5. Low variability and important factors

Including cognitive adjustments for low-variability and important factors involve reducing task difficulty, such as cultivating micro-habits, and enhancing positive energy through training in positive thinking. Also, according to the health theory of complementary and alternative therapy (Blue et al., 2016), some PhD students, for example, relieve stress by smoking and drinking. If they are not allowed to smoke, and there is no other alternative pastime to satisfy their inner needs, then this habit is difficult to change. Their bodies are used to this pattern and their subconscious will resist any change. However, if universities provide a good alternative, such as leading them in exercise, meditation or reading books, these methods can help people relax, relieve stress and better meet their inner needs. Therefore, it is also possible to cultivate new good habits to replace the original bad habits and to gradually consolidate these good habits in a long-term process. Therefore, for important but low-variable factors, a gradual substitution can be made on the basis of this theory.

### 2.3 Educational and ecological diagnosis: for self-evaluation and academic environment

The evolution of individual psychological self-consciousness is closely intertwined with external environmental changes. Research indicates that the combination of objective support, support utilization, and negative coping styles can collectively account for 18.6% of the variance in PhD students' mental health (Lu et al., 2012). Consequently, an education organization diagnosis is imperative to address PhD students' anxiety. ① Predisposing Factors: Predisposing factors primarily pertain to internal behavioral tendencies within individuals, focusing on the phenomenon of anxiety and individual consciousness. PhD students and their groups often exhibit vague cognition regarding the significance of reading (Liu, 2019), lack knowledge about stress and coping adjustments, and underestimate potential difficulties in the reading process. Insufficient confidence in overcoming challenges, confusion about future prospects, and pressure related to paper publishing and estimated graduation time further contribute to anxiety. This suggests a lack of judgment ability, metacognitive skills, and awareness of seeking external help. Additionally, the professional type and identity developed during undergraduate studies serve as predisposing factors for anxiety (Bazrafkan et al., 2016; Van Laethem et al., 2016). ② Enabling Factors: Enabling factors directly or indirectly influence the environment and the manifestation of target behaviors. PhD express anxiety about future career scenarios, including graduation, work prospects, and relationships (Wang et al., 2019). Current situations, such as the death of a loved one or papers not meeting graduation requirements, also impact their anxiety levels (Liu et al., 2020). The school's insufficient provision of resources for stress education and psychological counseling further hinders effective problem-solving. ③ Reinforcing Factors: Reinforcing factors play a crucial role in sustaining target behaviors by providing rewards or timely feedback. External social support, such as the “stocking” phenomenon among tutors, wherein they may overestimate the PhD students' abilities, and peer and family support, can serve as buffers against individual psychological distress, consequently reducing PhD students' anxiety levels (Sorrel et al., 2020). Recognizing and addressing these reinforcing factors is pivotal for creating a supportive academic environment.

### 2.4 Management policy assessment: for academic system and management policy

Given that educational issues are rooted in social systems, the development of educational policies necessitates careful consideration within these broader social contexts (Wang, 2016). The “PhD cultivation system” emerges as a critical factor contributing to PhD anxiety, directly translating into the phenomenon of “graduation pressure” for PhD students. Expectations from PhD student groups, families, universities, governments, and nations at large place significant demands on PhD education. The factors causing anxiety vary slightly among different PhD groups. For instance, the “Direct Ph.D. track from Master's” pathway presents unique academic pressures due to the shorter duration of academic training after the master's stage (Shi, 2019). Conversely, for Ph.D. students in medications, grappling with the frequent organization of information and the delicate balance between clinical work and scientific research, not only experience individual anxiety but also contribute to a broader phenomenon of “group anxiety” (Xu and Yang, 2020).

Furthermore, upon entering society, particularly within the realms of universities and research institutions, PhD students grapple with the challenges of socialization and adjustment. Exposure to occasional “negative information” in organizational management processes contributes to concerns about the future, leading to anticipatory avoidance and anxiety among PhD students. Addressing these multifaceted challenges requires a comprehensive assessment of existing management policies within the educational framework.

## 3 PROCEED stage: anxiety regulation and effects for PhD students

### 3.1 Implementation of targeted intervention program

For self-regulation: The targeted intervention program encompasses self-regulation, involving the application of individual stress management experiences and various common stress management techniques. Engaging in activities like talking with friends, eating, watching TV, socializing, exercising, sleeping, aromatherapy, drinking, shopping, smoking, massage, seeking treatment, meditation, yoga, and taking medication may offer only temporary relief from stress. Without addressing the underlying stressor, the sense of worry is likely to re-emerge soon (Bazrafkan et al., 2016). Existing research has not specifically focused on doctoral students utilizing exercise as a means to alleviate anxiety. Instead, studies have examined the general population, revealing that compared to inactive adults, those engaging in half the recommended amount of physical activity—equating to 4.4 marginal metabolic equivalent task hours per week (mMET-h/wk)—observed an 18% reduced risk of depression. Further, adults meeting the recommended physical activity threshold of 8.8 mMET-h/wk experienced a 25% lower risk of depression. The findings suggest that if less active adults had adhered to the current physical activity guidelines, approximately 11.5% of depression cases could have been averted (Pearce et al., 2022)). And for depression, exercise is effective in proportion to the intensity prescribed. Strength training and yoga seem to be the most acceptable modalities (Noetel et al., 2024).

For institution-assisted regulation: Major universities play a crucial role in providing institutional support for cognitive responses through professional psychological counseling services. These services encompass health education specifically tailored to manage anxiety, specialized psychological counseling, and workshop sessions that usually last between 6 and 8 weeks. The goal is to destigmatize psychological counseling and reduce the stigma associated with seeking mental health assistance. The content primarily focuses on stress management training, employing diverse methods, including rational emotive therapy, systematic desensitization, behavioral cognitive therapy (Liu, 2010), and integration with mindfulness meditation, yoga, and group discussions. Emphasis is placed on conveying to students the importance of perseverance in overcoming obstacles and the significance of self-forgiveness (Fan, 2017). The program aims to enhance students' understanding of the purpose of pursuing a PhD program, the challenges they might encounter, and the active role they play in fostering a positive relationship with their mentors. Additionally, students are encouraged to recognize the importance of self-care in the pursuit of a PhD degree (Hazell et al., 2020).

Universities can enhance course support capabilities: ① Offers training: Oxford University offers a specialized course titled “How to survive your PhD,” providing targeted support for PhD students navigating the challenges of their academic journey. ② Peer-Supported Learning Groups for Paper Writing: Recognizing the central role of paper writing, universities like Stanford have implemented supportive learning groups focused on peer support. Stanford's Supporting Writing Center organizes regular writing groups to directly enhance the writing skills of PhD candidates, addressing the core issue of anxiety. Writing camps, such as those at Stanford, set specific writing targets, such as requiring participants to produce at least 5,000 words in 3 days, offering a structured approach to alleviate procrastination (Fleming, 2019). ③ Popular “Time Management” Courses: Colleges and universities offer widely popular “time management” courses, catering to the specific needs of PhD students seeking effective strategies to balance their academic and personal commitments.

The management strategies include: ① For adjustment of PhD dissertation graduation criteria: Management policies can play a crucial role in supporting PhD students by appropriately adjusting graduation criteria for PhD dissertations (He et al., 2018). ② For teacher guidance and training oversight: Schools can regulate teacher guidance and oversee training to mitigate “stocking” behaviors among students. This involves gradually enhancing the academic atmosphere within the institution. Additionally, there may be adjustments to the university promotion system or clear industry planning to provide PhD students with increased clarity regarding their future prospects (Feng, 2012).

### 3.2 Process and impact evaluation

Self-regulation proves effective in reducing stress during the resistance period, with its primary purpose being the accumulation of strength. This stage is particularly suitable for PhD students in the alert and resistant stages, preventing their progression into the failure stage. By providing a buffer, individuals can step back, contemplate future challenges, reevaluate, and find solutions. Mild anxiety, commonly experienced by PhD candidates, is often resolved at this stage, positioning individuals in the “best alert period” for optimal results.

Institution-assisted regulation involves facilitating emotional expression, adjusting cognition, and providing timely referrals. This stage is beneficial for PhD students transitioning from the resistance stage to the failure stage. Challenges in this phase include the dependence on persistence for stress reduction, reluctance by some students to commit due to time constraints, fear of disclosure, and resistance to seeking psychological counseling based on cultural factors. The lack of a cognitive evaluation and selection mechanism leads to surface-level problem-solving through consultation, without addressing root causes. Additionally, a vicious circle may emerge, as high-anxiety individuals find it challenging to achieve self-forgiveness (Fan, 2017). Mechanism-assisted regulation aids in breaking this cognitive cycle, contributing to a reduction in anxiety levels.

Management Reform Assistance, such as the emphasis on foreign language articles for national project declarations, necessitates colleges and universities to enhance conditions for PhD graduation. The Ministry of Education has regulated tutor guidance for PhD, including specifying the time and frequency of guidance sessions (group meetings). This regulation supports the psychological and academic abilities of PhD students. Group interactions enable students to form peer groups for discussion, fostering a collaborative environment that helps alleviate anxiety during the scientific research process. Mentoring, both individual and in groups, provides crucial social support in the form of emotional, informational, and evaluative assistance. These mentoring groups create a non-competitive space where PhD students can share experiences, discuss challenges related to graduate school, laboratories, and career planning, thus enhancing their overall wellbeing.

### 3.3 Result evaluation

The evaluation tools employed in this study included a general conditions questionnaire and the Generalized Anxiety Disorder Scale (Xu and Yang, 2020), the Self-Rating Anxiety Scale (SAS) (Fan, 2017), and the PhD Psychological Pressure Self-Assessment Questionnaire (Guo, 2017). The assessment of anxiety was conducted using consistent measurement methods. Self-regulation demonstrated effectiveness in improving mild anxiety, while severe cases benefitted from cognitive-behavioral therapy and mindfulness-based stress reduction through institutional adjustment. Workshops such as the Anxiety Management Workshop and the Paper Writing Workshop yielded positive outcomes. For instance, a 5-day, 30-minute mindfulness meditation program, including 15 min of mindfulness practice (Noble et al., 2019), significantly improved students' ability to cope with stress, reduced anxiety symptoms, and enhanced academic focus. The attention level improvement post-training was also conducive to academic research concentration (Noble et al., 2019). The writing group's peer environment further provided robust social and emotional support (Guo, 2017).

Overall, literature indicates that positive emotions from blog reading outweigh negative emotions, with mentor guidance often leading PhD students from anxiety to positivity (Evans and Stevenson, 2011). Studies emphasize the significance of the “peer group” mode guided by mentors, providing PhD students with essential social support not necessarily available within the academic setting (Williams et al., 2017). Individual and institution-assisted regulation efforts have shown promise in alleviating PhD anxiety. However, studies also caution that while individuals may quickly recover from temporary stress, there may be “secondary effects” after the stress event (Van Laethem et al., 2017). Therefore, mentor guidance for PhD students should extend beyond scientific research support and psychological adjustment, fostering independence in research processes (Orer, 2020). This approach ensures that graduates can adapt to society and successfully conduct independent scientific research or other work post-graduation.

## 4 Conclusion and prospects

The current approach involves self-regulation, exercise, peer support, and medication. While these methods may provide some relief for up to 30% of the population, PhD students still face significant challenges related to anxiety, social support, and self-regulation. These challenges include poor adherence to exercise and medication regimens, mistrust of psychological counseling services, and inadequate coping skills in dealing with the pressures and challenges of graduation. Currently, it is common for PhD students to experience anxiety and require psychological support and problem-solving assistance. From an individual perspective, it is important to focus on self-regulating emotions and improving personal abilities to directly address stressors. From a university standpoint, adjusting graduation requirements, increasing scientific research and emotional adjustment courses, and providing reliable and privacy-protecting psychological counseling support are crucial. From a societal perspective, it is advisable to reduce the emphasis on the identity associated with a doctoral degree and implement policies that facilitate convenience in daily life. It is essential to cultivate an environment where pursuing a PhD and engaging in research are driven by genuine interest and aspiration, rather than a practical focus solely on obtaining a degree.

At the institutional level, facilitating an adaptive process between advisors and students is paramount. In cases where the advisor-student relationship adversely impacts a student's psychological well-being, institutions are encouraged to allow students to request a change of advisor.

The literature reviewed in this article primarily engages with case studies, qualitative research, and cross-sectional surveys, identifying only three interventional studies, all noted for their small sample sizes and non-RCT designs. For future research, it is advisable to extend the scope to include cross-cultural comparative analyses and longitudinal investigations. This expanded approach would enable a more nuanced understanding of the mental health challenges and influencing factors faced by PhD students, taking into account diverse cultural backgrounds and various stages of their academic journey.

Maintaining and enhancing the psychological well-being of PhD students requires more than just curricular training programs; it necessitates the support and transformative efforts of the organization. Hence, employing the PRECEDE-PROCEED model can enable researchers to identify a broader range of influencing factors and develop more comprehensive intervention strategies. As such, this approach not only highlights the importance of institutional support in fostering a healthy academic environment but also underscores the need for ongoing research and intervention development tailored to the unique challenges faced by PhD students. This paper advocates for a holistic strategy that integrates educational, organizational, and individual perspectives to effectively address and mitigate the psychological challenges of doctoral studies, paving the way for a healthier, more supportive academic journey.

## Author contributions

YM: Writing – original draft, Writing – review & editing. AY: Resources, Writing – review & editing. HM: Formal analysis, Writing – review & editing. YZ: Project administration, Writing – review & editing. XL: Validation, Writing – review & editing. HZ: Methodology, Writing – review & editing. YG: Methodology, Writing – review & editing.
