# Supplementary figures and images for: A narrative review of anxiety regulation in PhD students based on Green model
Source: Front Psychol. 2024 Jul 9;15:1351386. doi: 10.3389/fpsyg.2024.1351386 (PMC11264308; doi:10.3389/fpsyg.2024.1351386)

Supply Figure 1

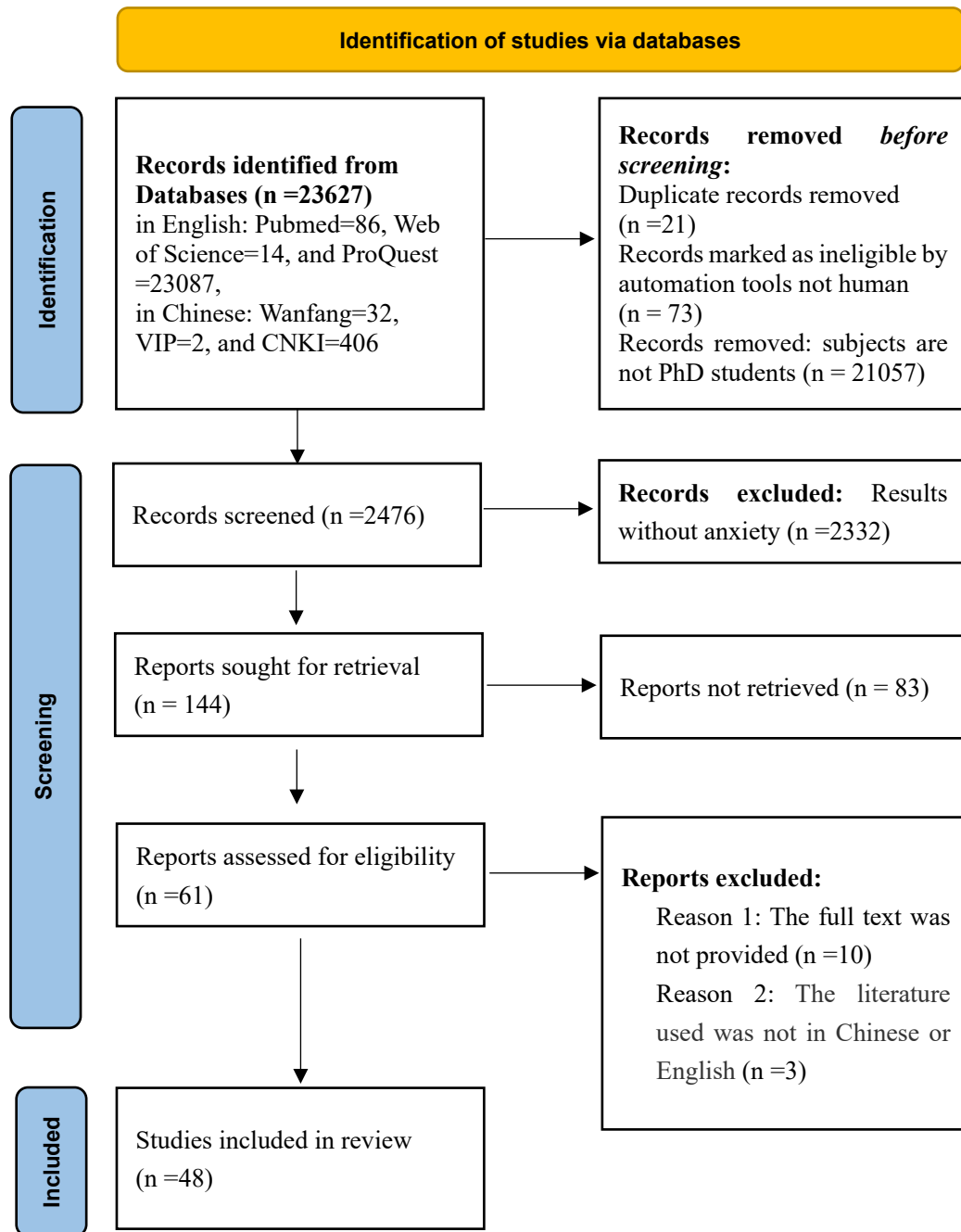

Supply Figure 1 PRISMA 2020 flow diagram for PhD students' anxiety

Supplement: Supplementary file 2 [file Image_1.pdf]
